# Psoriatic disease is associated with systemic inflammation, endothelial activation, and altered haemostatic function

**DOI:** 10.1038/s41598-021-90684-8

**Published:** 2021-06-22

**Authors:** Maria J. E. Visser, Chantelle Venter, Timothy J. Roberts, Gareth Tarr, Etheresia Pretorius

**Affiliations:** 1 Department of Physiological Sciences, Faculty of Science, Stellenbosch University, Private Bag X1 MATIELAND, Stellenbosch, 7602 South Africa; 2 Department of Biochemistry and Systems Biology, Institute of Systems, Molecular and Integrative Biology, Faculty of Health and Life Sciences, University of Liverpool, Liverpool, UK; 3 University College London Hospital NHS Foundation Trust, 250 Euston Road, London, NW1 2PB UK; 4 Division of Rheumatology, Institute of Orthopaedics and Rheumatology, Winelands Mediclinic Orthopaedic Hospital, Stellenbosch University, Cape Town, South Africa

**Keywords:** Biomarkers, Diseases, Rheumatology

## Abstract

Psoriasis is a chronic, immune-mediated inflammatory skin disease, affecting approximately 2% of the general population, which can be accompanied by psoriatic arthritis (PsA). The condition has been associated with an increased cardiovascular burden. Hypercoagulability is a potential underlying mechanism that may contribute to the increased risk of major cardiovascular events in psoriatic individuals. Whole blood samples were collected from 20 PsA patients and 20 healthy individuals. The concentrations of inflammatory molecules (C-reactive protein, serum amyloid A, soluble intercellular adhesion molecule-1, soluble vascular cell adhesion molecule-1, and soluble P-selectin) were determined by enzyme-linked immunosorbent assays. In addition, clotting efficiency was evaluated by thromboelastography. The fibrin network architecture was also assessed by scanning electron microscopy. Elevated levels of circulating inflammatory molecules were significantly associated with the presence of psoriatic disease. Furthermore, an increased tendency towards thrombus formation was significantly predictive of disease presence. Scanning electron microscopy revealed that fibrin clots were denser in psoriatic individuals, compared to healthy controls, with an increased fibrin fibre diameter associated with psoriatic disease. Our results add to the accumulating evidence of the systemic nature of psoriasis and the subsequent risk of cardiovascular comorbidities, potentially due to an acquired hypercoagulability. We suggest that haemostatic function should be monitored carefully in psoriatic patients that present with severe disease, due to the pre-eminent risk of developing thrombotic complications.

## Introduction

Psoriasis is a chronic, inflammatory skin disease, affecting approximately 2% of the global population^1^. Disease development is a complex interplay, involving genetic predisposition, environmental exposures, and disordered innate and adaptive immune responses. The condition commonly manifests as erythematous, well-demarcated plaques covered by slivery-white scales, and up to 30% of affected individuals could develop an inflammatory arthritis [psoriatic arthritis (PsA)]^2^. The cytokine network in psoriasis is primarily polarised towards the overexpression of T helper (T_H_) 1 and T_H_17 cytokines^3^. Prominent inflammatory mediators implicated in the initiation and maintenance of the disease, include interferon (IFN)-α, interleukin (IL)-22, the IL-23/IL-17 axis, and tumour necrosis factor (TNF)-α^4–7^. Chronic, subclinical systemic inflammation is evidenced by elevated levels of these and other inflammatory molecules in the blood of psoriatic patients. Individuals with psoriasis are also at an increased risk, when compared to the general population, of developing comorbidities such as depression, diabetes, inflammatory bowel disease, malignancy, metabolic syndrome, and cardiovascular disease (CVD)^8–13^. It has been proposed that shared inflammatory pathways may act as a driving force for both psoriasis and its extracutaneous manifestations^14^. Considering these observations, a paradigm shift has occurred from viewing psoriasis as merely ‘skin-deep’ to a systemic inflammatory condition^15^. A new concept, namely ‘psoriatic disease’, has also been introduced to describe skin and joint manifestations as well as the involvement of various other organ systems in the same individual^16^. Recently, the term ‘psoriatic syndrome’ has been proposed to rather define the condition as a syndrome that comprises diverse clinical features that may or may not occur at different stages of the disease^17^.

In recent years, CVD has been recognised as a prominent comorbidity of the condition. Various epidemiological studies have reported a significantly increased risk for major cardiovascular events, such as myocardial infarction (MI)^18^, stroke^19^, and venous thromboembolism (VTE)^8^, in individuals with psoriasis. Moreover, the presence of psoriasis has been identified as an independent risk factor for the development of CVD after adjusting for traditional risk factors^18–20^. Similarly, PsA also confers an increased risk for the development of cardiovascular events, such as MI and stroke^21^. It has been shown that the 10-year risk of adverse cardiac events, as determined by the Framingham Risk Score, is underestimated in these individuals^22^. Moreover, it has been recommended by the European League Against Rheumatism (EULAR) task force that CVD risk should be evaluated regularly in PsA patients, while also aiming to manage disease activity to lower the associated risk^23^.

Thrombophilia or hypercoagulability might be a potential mechanism underlying the relationship between psoriasis and CVD. The processes of inflammation and coagulation are interconnected, with these systems interacting in a bidirectional manner. A hallmark of sustained, low-grade systemic inflammation is a shift in the haemostatic balance towards a prothrombotic state^24^. Elevated levels of C-reactive protein (CRP), an acute-phase reactant and marker of inflammation, have been shown to be predictive of an increased risk of developing thrombotic disease^25–27^. Pro-inflammatory cytokines are the major mediators of inflammation-induced coagulation activation. These molecules disturb the haemostatic balance by inducing the expression of tissue factor (TF) on endothelial cells and monocytes, downregulating endogenous anticoagulant mechanisms, and impairing fibrinolytic activity^28,29^. TF plays a central role in coagulation, as it functions as a cofactor for factor VIIa in the extrinsic tenase complex. This complex initiates the extrinsic pathway of coagulation, resulting in the generation of thrombin. In the terminal stages of coagulation, soluble fibrinogen is converted into insoluble fibrin through the enzymatic action of thrombin. Fibrin plays a fundamental role in haemostasis, providing the structural scaffolding for blood clots. The fibrin network architecture is an important determinant of clot stability and fibrinolytic susceptibility^30^. Compact, less permeable clots, consisting of thin fibrin fibres, are associated with an increased risk of thrombotic events. This prothrombotic fibrin clot phenotype has been associated with various thromboembolic diseases, such as coronary artery disease^31^, stroke^32^, and VTE^33^. Therefore, characterisation of the fibrin network architecture might be a useful biomarker for thrombosis.

The vascular endothelium serves as an important interface between inflammation and coagulation, playing an essential role in the regulation of these entities. Under physiological conditions, the intact endothelium exhibits anti-inflammatory properties and expresses anticoagulant proteins^34,35^. However, upon stimulation with pro-inflammatory molecules, endothelial cells become activated^36^. Consequently, the endothelium upregulates the expression of cell adhesion molecules (CAMs) [intercellular adhesion molecule-1 (ICAM-1) and vascular cell adhesion molecule-1 (VCAM-1)], selectins (E-selectin and P-selectin), inflammatory mediators, and procoagulant factors, while attenuating the expression of anticoagulants. This functional deterioration of the endothelial barrier could exert a net prothrombotic effect. Additionally, the inflamed endothelium could promote the firm adhesion and full activation of platelets via the interactions between CAMs and/or selectins and platelet surface receptors^37,38^. The interaction of P-selectin with its receptor, P-selectin glycoprotein ligand-1, is a key mediator of endothelial-platelet interactions^39^ and the formation of platelet-leukocyte aggregates^37^. Impaired endothelial function and platelet hyperactivity have been implicated in the pathogenesis of arterial and venous thrombosis^40,41^.

In this paper, we investigated whether a hypercoagulable state is present in psoriatic patients, compared to healthy individuals. To this end, we evaluated whole blood (WB) coagulation efficiency and characterised the fibrin network architecture. In addition, the levels of biomarkers indicative of inflammation, endothelial dysfunction, and platelet activation were measured.

## Methods

### Ethical clearance and informed consent

This study received ethical approval from the Health Research Ethics Committee (HREC) of Stellenbosch University, Stellenbosch, South Africa (HREC reference N19/03/043). Prior to WB collection, a written form of informed consent was obtained from all study participants. All methods were carried out in accordance with the guidelines of the relevant ethics committees. We strictly adhered to the Declaration of Helsinki.

### Study design and study population

A cross-sectional study design was followed. The study population consisted of n = 40 volunteers, which included n = 20 PsA patients and n = 20 healthy individuals. Psoriatic patients were recruited during their visits to the Winelands Rheumatology Centre, Stellenbosch, for routine consultations. All psoriatic patients were assessed by the same rheumatologist. Psoriasis skin severity was assessed using the Psoriasis Area and Severity Index (PASI) score. For PsA, disease severity was assessed using the Disease Activity in PSoriatic Arthritis (DAPSA) score. According to DAPSA scores, PsA disease activity was classified as low, moderate, or high. Enthesitis was graded using the Leeds Enthesitis Index, while joint involvement was assessed by the 66/68 joint count for swollen and tender joints. Inclusion criteria for psoriatic patients were as follows: (1) fulfilled ClASsification of criteria for Psoriatic ARthritis (CASPAR) (see Table 1); (2) presence of any clinical variant of psoriasis; and (3) psoriatic patients were allowed to be on some form of systemic treatment. Corticosteroid usage included cortisone, methylprednisolone, and prednisone. Treatment with disease-modifying anti-rheumatic drugs (DMARDs) included leflunomide, methotrexate, and sulfasalazine. Biological agents included adalimumab (Humira), etanercept (Enbrel), infliximab (Revellex), and upadacitinib. Age-matched healthy individuals with no history of psoriasis or inflammatory disease were recruited. WB were collected at the Department of Physiological Sciences, Stellenbosch University, by a Medical Biological Scientist and phlebotomist registered with the Health Professionals Council of South Africa (MW: 0,010,782). Exclusion criteria for all study participants were as follows: (1) suffering from a known chronic inflammatory condition(s), namely human immunodeficiency virus, malignancies, and/or tuberculosis; (2) smoking; (3) using anticoagulant and/or antiplatelet medication; and (4) females using contraceptive medication or hormone replacement therapy.

**Table 1:** CASPAR criteria^75^ for the diagnosis of PsA.

Criterion	Description
1. **Evidence of psoriasis**	
Current psoriasis	Psoriatic skin or scalp disease as judged by rheumatologist or dermatologist
Personal history of psoriasis	History of psoriasis that may be obtained from patient, family physician, dermatologist, rheumatologist
Family history of psoriasis	History of psoriasis in first- or second-degree relative according to patient
2. **Psoriatic nail dystrophy**	Onycholysis, pitting, and hyperkeratosis
3. **Negative test for rheumatoid factor**	Determined by any method, except latex, but preferably by enzyme-linked immunosorbent assay or nephelometry; based on reference range of local laboratory
4. **Dactylitis**	
Current dactylitis	Swelling of entire digit
History of dactylitis	History as recorded by rheumatologist
5. **Radiographic evidence of juxtaarticular new bone formation**	Ill-defined ossification near joint margins (excluding osteophyte formation) on radiographs of hands or feet

A patient must have inflammatory articular disease (joint, spine, or entheseal) with a score of  ≥ 3 points of the following 5 criteria. Current psoriasis is assigned a score of 2, while all other features are assigned a score of 1.

### Collection of whole blood and preparation of platelet-poor plasma

A qualified nurse or phlebotomist collected WB from an antecubital vein via venipuncture, using standard sterile techniques. WB was collected in three 4.5 mL BD Vacutainer Citrate Tubes with 3.2% buffered sodium citrate solution (369714, Becton, Dickinson and Company, Franklin Lakes, NJ, USA,). Tubes were left at room temperature for at least 30 min before experiments were performed. Sample processing was completed within 24 h of blood collection. Platelet-poor plasma (PPP) was prepared by centrifuging WB at 3000 × *g* for 15 min at room temperature. Afterwards, PPP was aliquoted and stored at − 80 °C until further laboratory analysis.

### Thromboelastography

Thromboelastography (TEG) is a non-invasive viscoelastometric method which measures the ability of a blood sample to form a clot in a quantitative manner. TEG was performed, using the TEG 5000 Hemostasis Analyzer System (07-033, Haemonetics, Boston, MA, USA), to assess the clot kinetics and viscoelastic properties of naive (untreated) WB samples from psoriatic patients and healthy controls. Samples were prepared as follows: 20 μL of 0.2 M calcium chloride (CaCl_2_) (7003, Haemonetics) was added to a disposable TEG cup (6211, Haemonetics), followed by the addition of 340 μL of WB. CaCl_2_ was added to reverse the anticoagulant action of sodium citrate and consequently, activate the coagulation cascade. Samples were loaded in the measuring channels of the TEG and analyses were performed at 37 °C. Refer to Table 2 for a brief explanation of the seven TEG parameters that were assessed in this study.

**Table 2:** TEG clot parameters for WB^57,76,77^.

Parameter	Unit of measurement	Interpretation
Reaction time (R)	min	**Activation phase:** Time from start of test to first detectable fibrin formation (amplitude of 2 mm). Influenced by concentration of coagulation factors.
Kinetics (K)	min	**Amplification phase:** Time taken to form a clot with a certain level of strength (amplitude of 20 mm). Influenced by fibrinogen concentration and to a lesser extent, platelet function.
Alpha angle (A)	deg	**Propagation phase:** The angle measures the maximal speed of thrombin generation and fibrin formation and cross-linking. Influenced by fibrinogen concentration and to a lesser extent, platelet function.
Maximum amplitude (MA)	mm	**Termination phase:** Maximum mechanical strength/stiffness of clot. Influenced by fibrin cross-linking, platelet count and platelet glycoprotein IIb/IIIa interactions.
Maximum rate of thrombus generation (MRTG)	dynes/cm^2^/s	First derivative of the velocity of the increase in clot strength based on the change in the elastic modulus, G, where G = (5000MA)/(100-MA).
Time to maximum rate of thrombus generation (TMRTG)	min	Time interval observed before maximum speed of clot growth.
Total thrombus generation (TTG)	dynes/cm^2^	Total area under the velocity curve, representing the clot strength generated during clot growth.

### Scanning electron microscopy

Fibrin clots were prepared from PPP for ultrastructural analysis of fibrin fibres by scanning electron microscopy (SEM). To create a fibrin fibre network, 10 μL of PPP was deposited on a 10 mm round glass coverslip and 5 μL of human thrombin (provided by the South African National Blood Service, final concentration 7 IU/mL) was added. Subsequently, samples were covered with 1X Gibco phosphate-buffered saline (PBS), pH 7.4 (10010015, Thermo Fisher Scientific, Waltham, MA, USA) for at least 15 min. Afterwards, primary fixation was performed by covering samples with 4% formaldehyde (158127, Sigma-Aldrich, St. Louis, MO, USA), which cross-links proteins, for a minimum of 30 min. Thereafter, samples were washed three times for 3 min with PBS. Secondary fixation was performed with 1% osmium tetra-oxide (OsO_4_) (75632, Sigma-Aldrich), which cross-links lipids, for 15 min. Afterwards, samples were washed three times for 3 min with PBS. Samples were serially dehydrated with increasing concentrations of ethanol, 30%, 50%, 70%, 90% and three times with 100% ethanol, for 3 min each. A dehydration step was performed by covering samples with 99.9% hexamethyldisilazane (HMDS) (379212, Sigma-Aldrich) for 30 min. HMDS was removed and a final drop of HMDS was added directly to the sample, whereafter samples were left to air-dry overnight in a flow-hood. Coverslips were mounted with double-sided carbon tape on glass microscope slides and sputter coated with carbon, using a Quorum Q150T E Plus carbon coater (Quorum Technologies, Laughton, East Sussex, UK). Samples were examined using the Zeiss MERLIN Field Emission Scanning Electron Microscope (Carl Zeiss, Oberkochen, Germany), housed at the Central Analytical Facilities Electron Microscopy Unit, Stellenbosch University. Electron micrographs were captured with the high resolution InLens detector at 1 kV.

**Figure 1:**
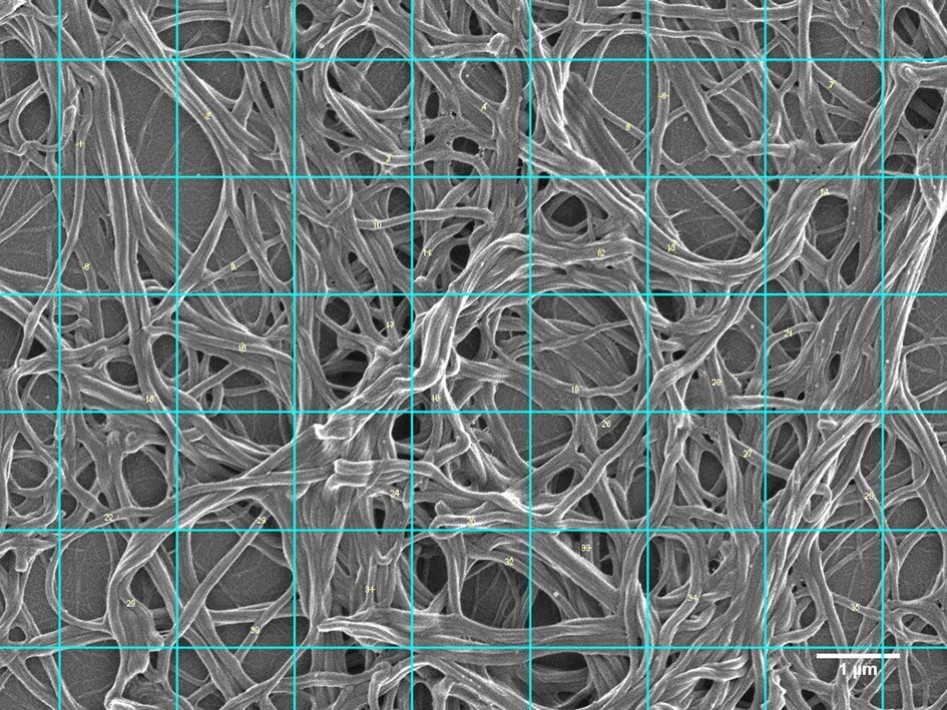
Representative scanning electron micrograph with grid overlay. Numbered fibres indicate fibres that were measured. Individual grid blocks represent an area of 2 μm^2^. Total grid area is 97 μm^2^ (scale bar = 1 μm).

Micrographs of a subset of psoriatic patients (n = 9) and healthy controls (n = 9) were identified for further analysis of fibrin fibre diameter, using ImageJ (version 1.52a). A grid was overlaid on these micrographs, consisting of 5 vertical blocks and 7 horizontal blocks. Each 1.4 μm × 1.4 μm grid block measured an area of 2 μm^2^. In each block of the grid, a fibrin fibre was selected randomly, and the fibre diameter measured (Fig. 1). For each of the individuals included in the subset, three micrographs were analysed, with 35 fibres measured per micrograph. Therefore, 105 measurements were taken per individual. In total, 945 measurements were taken in both the patient and control groups. In order to graphically illustrate differences in the distribution of fibrin fibre diameter, in healthy controls and psoriatic patients, frequency bar graphs were constructed. Additionally, differences in the minimum, maximum, and mean fibre diameter were evaluated between groups.

### Soluble P-selectin

The concentration of soluble (s)P-selectin/CD62P was determined using the Human sSELP (Soluble P-Selectin) ELISA kit (EH3818, Fine Biotech Co., Ltd., Wuhan, Hubei, China). Prior to analysis, PPP from psoriatic patients and healthy controls were thawed from − 80 °C to room temperature. PPP was diluted 250X with the supplied Sample Dilution Buffer. Before samples were added, the supplied ELISA microplate (pre-coated with capture antibodies) was washed two times with Wash Buffer. Subsequently, 100 μL of sample, standard or control was added per well and incubated for 1.5 h at 37 °C. After incubation, the plate was washed two times with Wash Buffer. Thereafter, 100 μL of biotin-labelled antibody was added per well and incubated at 37 °C for 1 h. The plate was washed again three times with Wash Buffer. Subsequently, 100 μL of the horseradish peroxidase (HRP)-streptavidin conjugate was added per well and incubated for 30 min at 37 °C. Streptavidin binds to biotin with high affinity. After washing the plate five times with Wash Buffer, 90 μL of 3,3′,5,5′-tetramethylbenzidine (TMB) substrate was added per well and incubated in the dark for 10 min at 37 °C. TMB is a colorimetric substrate which reacts with HRP. Finally, 50 μL of Stop Solution was added per well, resulting in the formation of a yellow reaction product. The absorbance was read at a wavelength of 450 nm. Samples were analysed in duplicate.

### Vascular injury panel

Biomarker analysis was performed using the V-PLEX Vascular Injury Panel 2 (human) Kit (K15198D, Meso Scale Diagnostics, Rockville, MD, USA). This kit measures four biomarkers associated with acute inflammation and tissue damage, namely CRP, serum amyloid A (SAA), soluble (s)ICAM-1/CD54, and soluble (s)VCAM-1/CD106. Prior to analysis, PPP from psoriatic patients and healthy control subjects were thawed from − 80 °C to room temperature. PPP was diluted 1000X with the supplied MSD Diluent, as recommended by the manufacturer. Before samples were added, the supplied MSD MULTI-SPOT 96-Well Spot plate was washed three times with MSD Wash Buffer. Subsequently, 25 μL of sample, calibrator or control was added per well (precoated with capture antibodies) and incubated for 2 h at room temperature. After incubation, the plate was washed again three times with MSD Wash Buffer. Thereafter, 25 μL of detection antibody was added per well and incubated for 1 h. Detection antibodies (MSD SULFO-TAG) are conjugated with electrochemiluminescent labels. The plate was washed one final time with MSD Wash Buffer and 150 μL of MSD Read Buffer was added to each well. The plate was read on the MSD Discovery Workbench 4 instrument by applying a voltage, which causes the emittance of light. The intensity of emitted light is proportional to the amount of analyte present in the sample. The MSD DISCOVERY WORKBENCH software was used to acquire and analyse data. Samples were analysed in duplicate.

### Statistical analysis

R version 4.0.3 was used to perform logistic regression to determine the strength of associations between study variables and disease status (presence or absence of psoriatic disease). More specifically, logistic regression was performed on covariates directly (Model 1) and with adjustment for age and gender (Model 2). Odds ratios (ORs) are reported with 95% confidence intervals (CIs). GraphPad Prism version 8.4.3 was used to produce summaries and plots of the data. In order to summarise differences between groups, an unpaired t-test was performed for normally distributed data (as determined by the Shapiro Wilk normality test), while the Mann–Whitney test was performed for non-normally distributed data. Normally distributed data is expressed as mean ± standard error of the mean and non-normally distributed data is expressed as median and (25–75% quartile range). A *p*-value of less than 0.05 was considered to be statistically significant. A single missing TEG K value was imputed, using the mean of the K values of the control population.

## Results

Demographic features and clinical characteristics of healthy individuals and psoriatic patients are presented in Table 3. There were no significant differences in the age and gender between the study groups. According to DAPSA scores, 4 patients presented with low disease activity, 7 with moderate disease activity, and 9 with high disease activity. Dactylitis was present in 7 patients. The median PASI score was 5.300, representing moderate disease severity.

**Table 3:** Demographic features and clinical characteristics of healthy individuals and psoriatic patients.

Demographics	Healthy individuals (n = 20)	Psoriatic patients (n = 20)
Age, years	58.15 ± 2.785	54.25 ± 2.148
**Gender**		
Female, n (%)	6 (30)	8 (40)
Male, n (%)	14 (70)	12 (60)
**Comorbidities**		
Anaemia, n (%)		1 (5)
Diabetes, n (%)		5 (25)
Hypercholesterolaemia, n (%)		4 (20)
Hypertension, n (%)		9 (45)
Hypothyroidism, n (%)		2 (10)
Ischaemic heart disease, n (%)		3 (15)
Menopause, n (%)		1 (5)
**Disease activity**		
DAPSA		
Low, n (%)		4 (20)
Moderate, n (%)		7 (35)
High, n (%)		9 (45)
Dactylitis, n (%)		7 (35)
PASI		5.300 (1.075–14.85)
**Treatment**		
Topical corticosteroids, n (%)		3 (15)
DMARDs, n (%)		12 (60)
Biologic agent, n (%)		4 (20)

Normally distributed data is expressed as mean ± standard error of the mean and non-normally distributed data is expressed as median and (25–75% quartile range).

**Figure 2:**
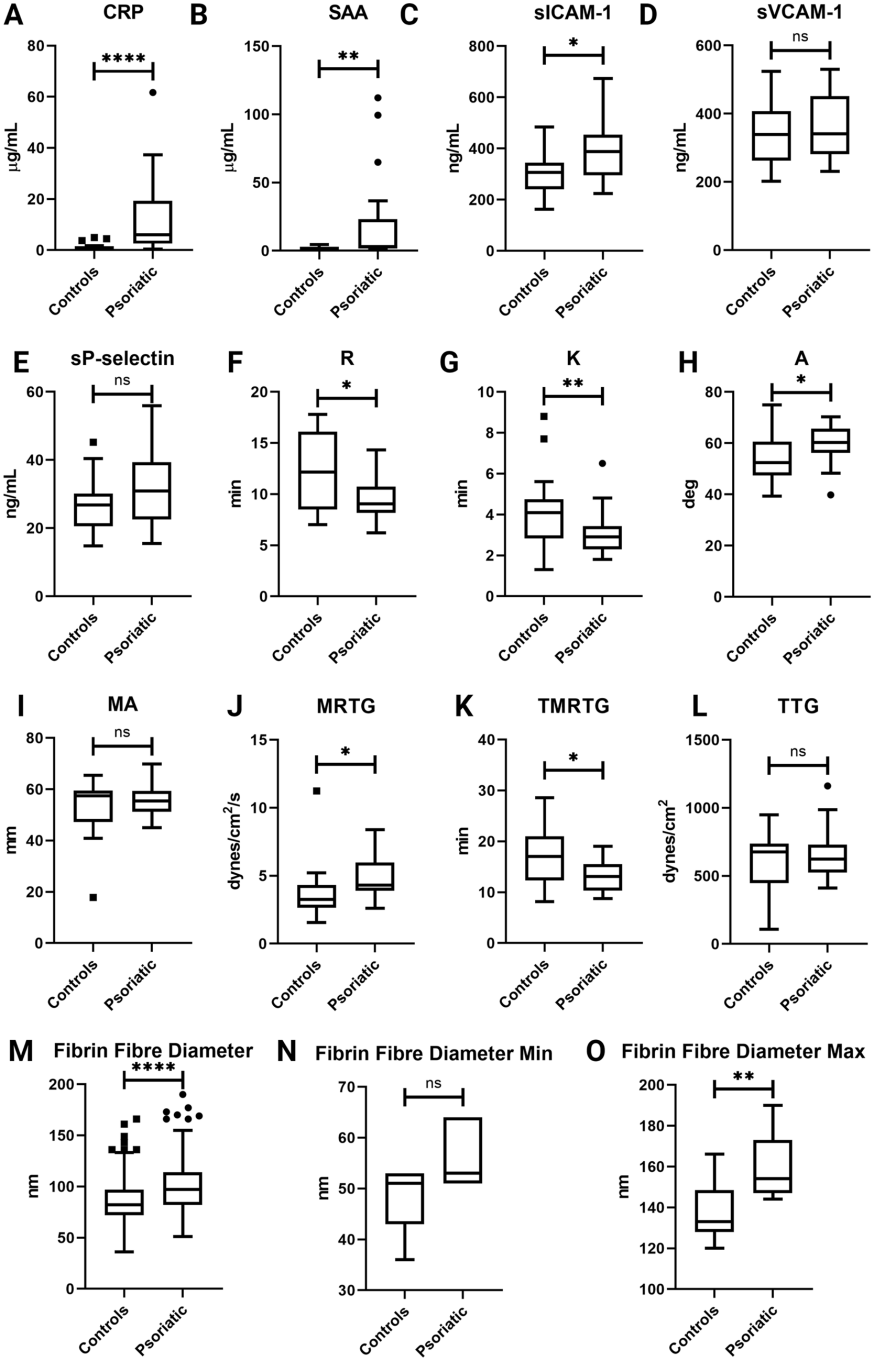
Box-and-whisker plots illustrating the distribution of parameters assessed in this study in healthy individuals and psoriatic patients. (**A**–**D**) indicates V-PLEX panel results, (**E**) indicates sSELP ELISA results, (**F**–**L**) indicates TEG results, and (**M**–**O**) indicates fibrin fibre diameter results. Asterisks indicate statistically significant differences between the groups, as determined by either an unpaired t-test or Mann–Whitney test, where ****p < 0.0001, ***p < 0.001, **p < 0.01, and *p < 0.05. Box-and-whisker plots were produced using GraphPad Prism version 8.4.3.

Biomarker analysis was performed to determine the levels of specific inflammatory molecules in the PPP of healthy individuals and psoriatic patients. Box-and-whisker plots, showing the distribution of these parameters in healthy individuals and psoriatic individuals, are shown in Fig. 2. CRP, SAA, and sICAM-1 levels were significantly elevated in the psoriatic group when compared to controls (Fig. 2). No significant differences were noted in the sVCAM-1 levels when psoriatic individuals were compared to control subjects. Logistic regression models indicated that elevated levels of acute-phase reactants, namely CRP (OR 1.402 CI 1.146–1.838) and SAA (OR 1.144 CI 1.038–1.315), were significantly associated with the presence of psoriatic disease (Table 4). Elevated concentrations of these biomarkers indicate the presence of inflammation in psoriatic patients. In addition, sICAM-1 (OR 1.012 CI 1.003–1.022) was another marker significantly associated with psoriatic disease (Table 4). Raised levels of sICAM-1 reflect endothelial cell activation. After adjusting for the effects of age and gender, higher sP-selectin (OR 1.101 CI 1.005–1.228) levels were significantly associated with disease presence (Table 4). An elevated concentration of sP-selectin indicates platelet activation, as this adhesion molecule is expressed on the platelet surface upon activation.

**Table 4:** Results of logistic regression on laboratory parameters in healthy individuals and psoriatic patients.

Inflammatory markers	Healthy individuals (n = 20)	Psoriatic patients (n = 20)	Unadjusted OR (95% CI)	Adjusted OR (95% CI)
CRP (µg/mL)	0.57 (0.295–1.508)	6.015 (2.565–19.082)	1.375 (1.145–1.724)*	1.402 (1.146–1.838)*
SAA (µg/mL)	1.11 (0.59–2.615)	2.66 (2.02–15.332)	1.145 (1.034–1.320)*	1.144 (1.038–1.315)*
sICAM-1 (ng/mL)	306.15 (270.36–342.47)	388.48 (314.75–449.47)	1.009 (1.002–1.019)*	1.012 (1.003 –1.022)*
sVCAM-1 (ng/mL)	338.94 (279.42–400.59)	340.93 (297.68–449.79)	1.003 (0.996–1.011)	1.005 (0.997–1.014)

Platelet activation marker	Healthy individuals (n = 15)	Psoriatic patients (n = 20)	Unadjusted OR (95% CI)	Adjusted OR (95% CI)
sP-selectin (ng/mL)	26.78 (22.825–29.34)	30.86 (23.873–39.013)	1.064 (0.987–1.159)	1.101 (1.005–1.228)*

Data is expressed as median and (25–75% quartile range). ORs are reported at 95% CIs. Asterisks indicate statistically significant associations.

TEG was performed on WB samples from healthy controls and psoriatic patients to assess coagulation sufficiency. Seven WB clot parameters were assessed in this study (Table 2) and the distribution of TEG parameters are illustrated by box-and-whisker plots in Fig. 2. Significant differences were detected in five TEG parameters (Fig. 2 and Table 5). Logistic regression modelling showed that shortened R (OR 0.682 CI 0.497–0.877) and K (OR 0.384 CI 0.169–0.733) values were significantly associated with psoriatic disease. Accelerated fibrin cross-linking, indicated by an increase in A (OR 1.157 CI 1.043–1.302), was also identified as a significant parameter. Moreover, after adjustment, an increase in the MRTG (OR 1.637 CI 1.046–2.894) was also significantly associated with psoriatic disease. Furthermore, a significant association was also detected between a shortened TMRTG (OR 0.778 CI 0.636–0.922) value and disease presence. Altogether, these results indicate that an altered coagulation profile, characterised by an increased tendency to form a blood clot, is associated with psoriatic disease.

**Table 5:** Results of logistic regression on TEG WB clot parameters in healthy individuals and psoriatic patients.

TEG WB clot parameter	Healthy individuals (n = 20)	Psoriatic patients (n = 20)	Unadjusted OR (95% CI)	Adjusted OR (95% CI)
R (min)	12.15 (8.7–15.975)	9.05 (8.25–10.575)	0.732 (0.555–0.919)*	0.682 (0.497–0.877)*
K (min)	4.095 (2.875–4.65)	2.9 (2.5–3.275)	0.490 (0.242–0.816)*	0.384 (0.169–0.733)*
A (deg)	52.45 (48.05–60.525)	60.25 (57.325–63.825)	1.112 (1.021–1.224)*	1.157 (1.043–1.302)*
MA (mm)	57.45 (49.125–58.9)	55.4 (52.25–59.4)	1.048 (0.976–1.145)	1.055 (0.969–1.178)
MRTG (dynes/cm^2^/s)	3.255 (2.758–4.303)	4.315 (3.918–5.963)	1.530 (0.993–2.537)	1.637 (1.046–2.894)*
TMRTG (min)	17.05 (12.397–20.94)	13.125 (10.397–15.52)	0.821 (0.677–0.954)*	0.778 (0.636–0.922)*
TTG (dynes/cm^2^)	676.95 (483.79–719.4)	623.84 (547.41–717.96)	1.002 (0.998–1.005)	1.002 (0.998–1.006)

Data is expressed as median and (25–75% quartile range). ORs are reported at 95% CIs. Asterisks indicate statistically significant associations.

**Figure 3:**
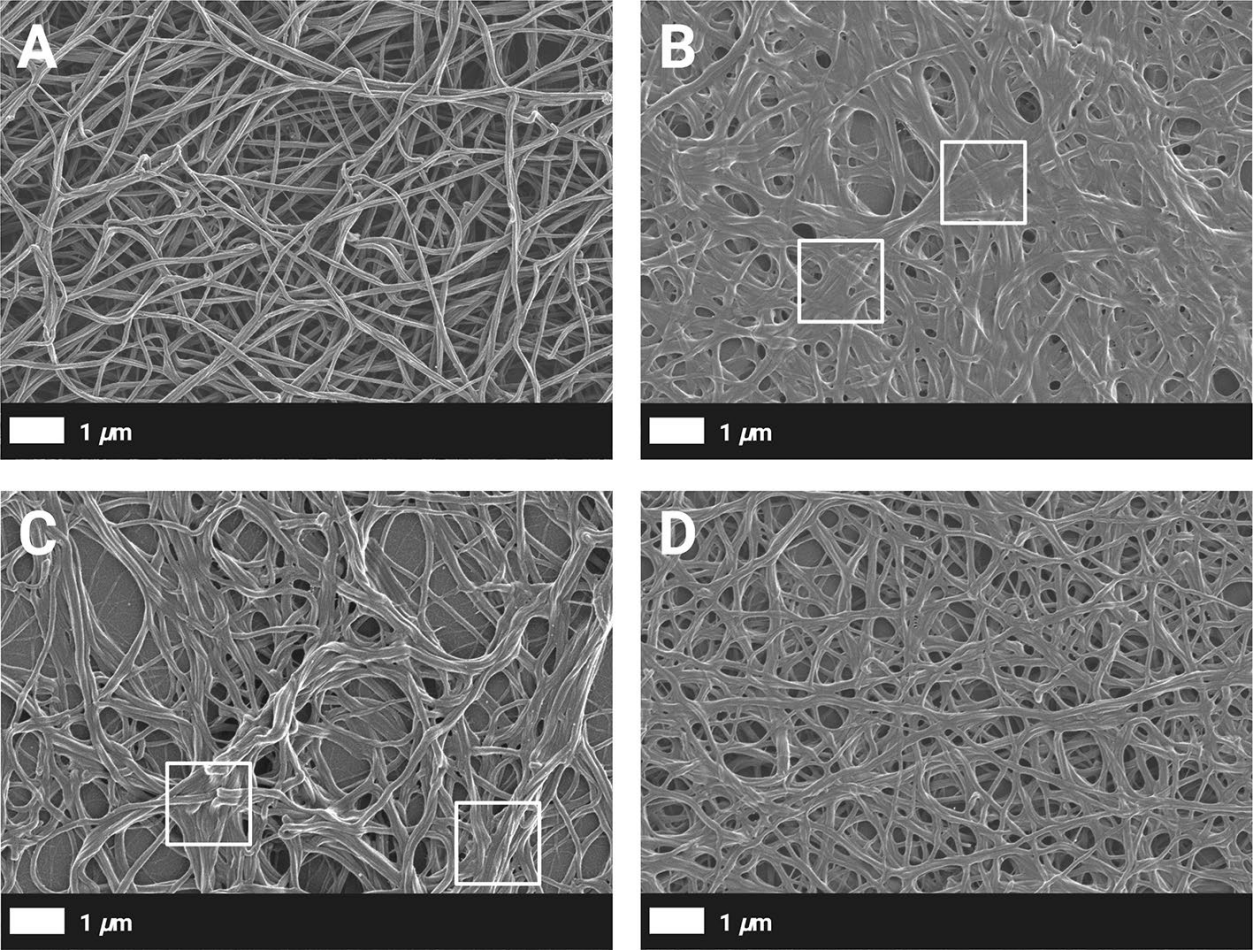
(**A**–**D**) Representative scanning electron micrographs of fibrin networks (prepared form PPP) from a healthy individual and psoriatic patients. (**A**) A typical fibrin network of a healthy 47-year-old female individual. (**B**–**D**) Fibrin networks of psoriatic individuals. (**B**) A fibrin network of a 45-year-old female individual with low PsA activity and mild skin involvement. (**C**) A fibrin network of a 40-year-old male individual with moderate PsA activity and mild skin involvement. (**D**) A fibrin network of a 49-year-old male individual with high PsA activity and moderate skin involvement.

The fibrin network architecture may influence clot properties; therefore, SEM was utilised to detect differences in the ultrastructure of fibrin fibre networks (PPP clots) from healthy individuals and psoriatic individuals. Representative scanning electron micrographs of PPP clots of a healthy control and psoriatic patients are depicted in Fig. 3A–D. Typically, a fibrin network from a healthy individual (shown in Fig. 3A) appeared as a ‘loose’ network of fibrin fibres. Individual fibres were clearly discernible, and pores were observed regularly between fibres. In contrast, fibrin networks of psoriatic individuals seemed to be more compact (Fig. 3B–D). Individual fibrin fibres could not always be distinguished, and multiple fibres were fused (indicated by the boxes in Fig. 3B, C).

**Figure 4:**
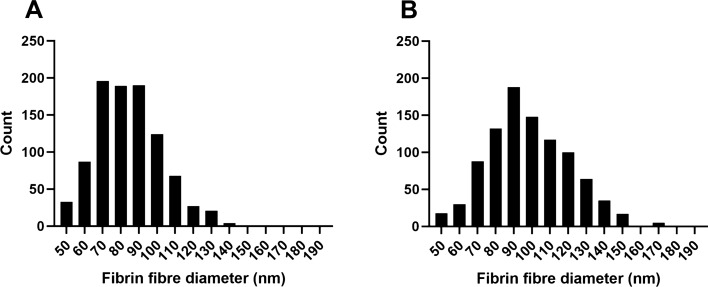
Frequency bar graphs of fibrin fibre diameter distribution. (**A**) indicates healthy individuals and (**B**) indicates psoriatic patients. Bar graphs were produced using GraphPad Prism version 8.4.3.

Fibrin fibre diameter was measured in a subset of healthy individuals and psoriatic individuals. Psoriatic patients included in the subset were chosen to represent low, moderate, and high disease activity of PsA (according to DAPSA scores), with mild to moderate skin involvement (according to PASI scores), to ensure that disease activity of both the joint and skin domains were taken into account. The mean, minimum, and maximum fibre diameter were determined for each individual in both the control and patient groups. For the minimum and maximum fibre diameter, the smallest measurement and largest measurement of an individual were grouped as follows: minimum of healthy individuals, maximum of healthy individuals, minimum of psoriatic patients, and maximum of psoriatic patients. Figure 2 shows box-and-whisker plots illustrating the distribution of these variables in the study populations. Frequency bar graphs were constructed, as indicated in Fig. 4, to graphically illustrate differences in the distribution of fibrin fibre diameter in healthy individuals and psoriatic patients. In comparison to healthy controls, the fibrin fibre diameter was significantly increased in psoriatic individuals (Fig. 2). This was also reflected by the frequency bar graphs which show a greater count of thicker fibres in psoriatic patients (Fig. 4B). Logistic regression analysis showed that an increased fibrin fibre diameter (OR 1.562 CI 1.234–2.173) was a significant predictor of disease presence (Table 6). Similarly, an increase in the minimum (OR 1.483 CI 1.126–2.225) and maximum fibrin fibre diameter (OR 1.121 CI 1.041–1.240) were also associated with the presence of psoriatic disease (Table 6).

**Table 6:** Results of logistic regression on fibrin fibre diameter in healthy individuals and psoriatic patients.

Fibrin fibre diameter	Healthy individuals (n = 9)	Psoriatic patients (n = 9)	Unadjusted OR (95% CI)	Adjusted OR (95% CI)
Mean (nm)	83.724 (83.39–86.552)	99.043 (93.336–106.12)	1.470 (1.172–2.014)*	1.562 (1.234–2.173)*
Minimum mean (nm)	51 (46–53)	53 (51–62.25)	1.410 (1.107–2.062)*	1.483 (1.126–2.225)*
Maximum mean (nm)	133 (130–136)	160 (150–168.25)	1.112 (1.036–1.230)*	1.121 (1.041–1.240)*

Data is expressed as median and (25–75% quartile range). ORs are reported at 95% CIs. Asterisks indicate statistically significant differences.

## Discussion

**Figure 5:**
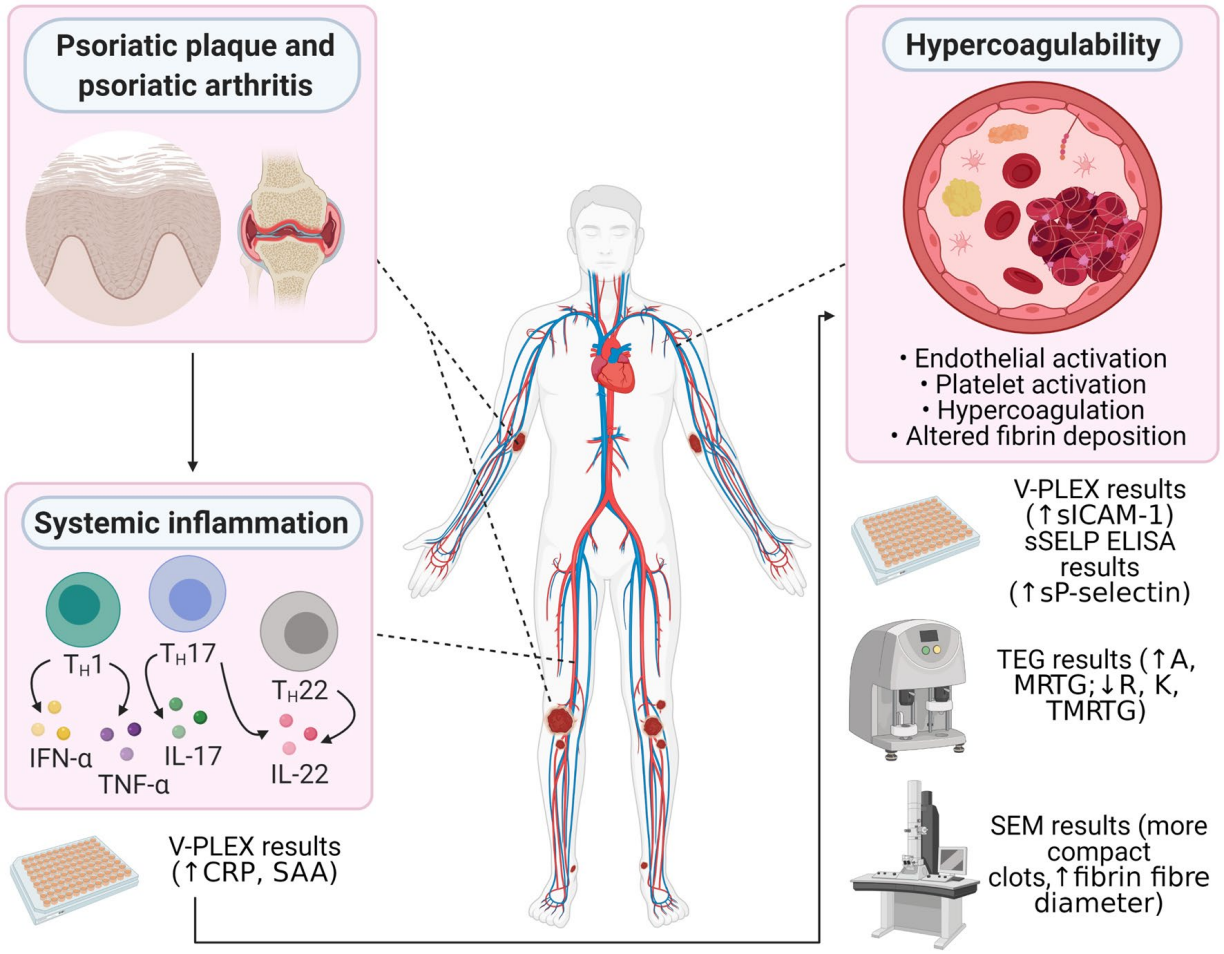
A summary of the key findings of this study. Various pro-inflammatory molecules are implicated in the onset and maintenance of psoriatic disease. These inflammatory mediators may spill over into circulation, resulting in systemic inflammation. Accordingly, psoriatic patients presented with elevated levels of acute-phase reactants (CRP and SAA). As the processes of inflammation and coagulation are interconnected, persistent systemic inflammation may promote the development of a prothrombotic state in psoriatic individuals. In this study, the prothrombotic state in psoriatic patients were characterised by endothelial (elevated sICAM-1 levels) and platelet activation (elevated sP-selectin levels), hypercoagulability (TEG results), and abnormal fibrin deposition (SEM analysis). Diagram created with BioRender.com.

Psoriasis is a T-cell mediated chronic, inflammatory skin disease characterised by the hyperproliferation of keratinocytes. However, several extracutaneous manifestations have also been linked with the condition^42,43^. In particular, an increased burden of cardiovascular morbidity and mortality has been observed in psoriatic individuals. Nevertheless, the causes responsible for CVD prevalence in psoriatic disease have not been fully elucidated. Thrombotic complications, such as MI^19,21^, stroke^18,21^ and VTE^8^, have been shown to occur more frequently in psoriatic individuals. In the present study, we show that psoriatic patients presented with a biomarker profile that reflected systemic inflammation, endothelial activation, and heightened platelet activity. Moreover, altered viscoelastic properties of WB as well as structural changes in the fibrin network ultrastructure were observed in these individuals. See Fig. 5 for a summary of results.

The presence of a peripheral inflammatory milieu in psoriatic patients was confirmed by the association between increased levels of CRP and SAA and the condition (Table 4). These proteins are acute-phase reactants which are hepatically synthesised and released into circulation in response to inflammation and/or tissue injury. TNF-α, a major mediator of psoriasis pathogenesis, has been shown to induce the expression of IL-6. This cytokine, which is also produced by keratinocytes in psoriatic skin lesions^44^ and the inflamed synovium in PsA^45^, stimulates the synthesis of CRP. Therefore, elevated CRP levels may reflect the participation of pro-inflammatory cytokines in the psoriatic disease process. SAA is also overexpressed by epidermal keratinocytes in lesional skin of psoriatic patients^46^ and may also be detected in the inflamed synovial tissue of PsA patients^47^. Both CRP and SAA possess procoagulant activity and may contribute to a hypercoagulable state via the induction of TF in endothelial cells and monocytes as well as the suppression of anticoagulant activity^48–52^. Furthermore, endothelial cell activation, indicated by raised sICAM-1 levels, was also associated, albeit weakly, with the presence of disease (Table 4). Membrane-bound ICAM-1 is involved in leukocyte extravasation to sites of inflammation^53^, and the overexpression of this CAM has been demonstrated in lesional and non-lesional skin of psoriatic individuals^54^. ICAM-1 may promote thrombus formation by facilitating the adhesion of activated platelets to the endothelium^55,56^. One may speculate that the increased plasma levels of the aforementioned molecules are due to a ‘spillover’ effect from affected skin areas, reinforcing peripheral inflammation.

Limited studies have investigated the coagulation profile of psoriatic individuals. We assessed blood coagulation using TEG, as it provides a more comprehensive view of coagulation status compared to conventional laboratory tests^57^. Shortened values of parameters that measure clot formation initiation and propagation (R, K) were associated with the presence of disease (Table 5). Enhanced clot propagation (A, MRTG, TMRTG) was also related to the presence of psoriatic disease (Table 5). These changes may be attributed to elevated levels of fibrinogen or an increased rate of thrombin generation. We did not measure fibrinogen levels in this study; however, other studies have reported raised levels in psoriatic patients^58–60^. Pro-inflammatory molecules, which circulate at elevated levels in active psoriasis, may induce TF expression by vascular endothelial cells and peripheral blood monocytes^61^ while simultaneously suppressing the activity of thrombomodulin^62^, leading to rapid thrombin generation and fibrin deposition. Despite an increased tendency to form a clot, parameters related to clot strength (MA, TTG) appeared unaltered in psoriatic individuals compared to healthy individuals (Table 5). This finding was likely caused by compromised fibrin network structure and/or platelet function in psoriatic patients. It should be noted that several patients that were included in this study received some form of immunosuppressant therapy (Table 3), which may be a confounder. Methotrexate and biological agents may potentially cause thrombocytopenia^63^. On the contrary, various studies have reported that platelets are in an activated state in psoriatic patients^64–66^. In the present study, platelets did appear to be activated as elevated sP-selectin levels were associated with psoriatic disease (Table 4). Importantly, refractoriness of platelets (as a result of hyperactivation) may decrease their ability to participate in the process of clot contraction, which may also influence the outcome of an occlusive thrombus. Tutwiler and colleagues (2017) have reported impaired clot contraction in acute ischaemic stroke patients, suggesting that this may lead to a greater reduction in intravascular blood flow^67^. A more recent study, by the same group, also notes that diminished clot contraction may be predictive of embolisation in pathological states^68^.

Altered fibrin network architecture have been linked to the development of thrombotic disease^69^, therefore, we assessed plasma clot ultrastructure with SEM. To our knowledge, this is the first study that has examined the fibrin network structure in psoriatic patients. In the current study, the fibrin network of psoriatic individuals appeared to be denser with various fused fibres (Fig. 3B–D). An increased fibrin fibre diameter (across all groups) was also more prevalent in psoriatic patients (Table 6). It has been reported that denser clots composed of thin fibrin fibres are more resistant to fibrinolytic degradation, than clots that are composed of thick fibres^31^. However, it should be kept in mind that various factors, such as the concentration of coagulation factors (*e.g.* thrombin^70^), cellular interactions (*e.g.* neutrophil extracellular trap formation^71^), and posttranslational modifications (*e.g*. oxidation^72^), may determine final clot structure. We hypothesise that the altered structural properties of fibrin clots along with an increased propensity to form a clot (as indicated by TEG results), may confer an increased risk for thrombosis in psoriatic patients. In addition, these patients may potentially be more susceptible to embolisation, as platelet function seems to be impaired either due to hyperactivation, or a secondary effect of systemic therapy.

Our study has limitations. This study is cross-sectional in nature; therefore, no inferences can be made with regards to causality. In addition, the number of study participants was limited. Finally, psoriatic patients receiving systemic treatment were included in this study, which may have a potentially confounding effect. Further studies are required to confirm our findings.

## Conclusion

Psoriatic disease is associated with an increased risk to develop CVD. Here, we confirm that circulating inflammatory molecules are significantly increased in our disease population. Our results therefore add to the accumulating evidence of the systemic inflammatory nature of psoriasis and the subsequent risk of cardiovascular comorbidities. We also show that psoriatic patients present with an altered coagulation profile, defined by an inclination towards thrombus formation. Additionally, we also demonstrate for the first time, that denser fibrin networks composed of thick fibrin fibres are formed in psoriatic individuals. These changes might have implications for the outcome of thromboembolic complications in the context of psoriatic disease. Future prospective studies should be conducted to confirm our findings and address remaining questions, such as determining the lytic susceptibility of an altered clot structure in psoriatic disease. We suggest that haemostatic function should be monitored carefully in psoriatic patients that present with severe disease and/or inflammatory flares, due to the pre-eminent risk of thrombotic complications. We recommend the routine monitoring of coagulability by a global coagulation assay, such as TEG, not as a predictor of imminent thrombotic risk, but rather as a preventative measure. Levels of fibrinogen and D-dimer may also be considered for this purpose, as they show strong prognostic value for the development of thrombotic events^73,74^.

## Data Availability

The datasets generated during and/or analysed during the current study are available in the Onedrive Blood Laboratory Repository at https://1drv.ms/u/s!AgoCOmY3bkKHiv9WfY1-yYCV8Bl5Iw?e=wYMG5N.
